# Bio-ceramics application in Dentistry

**DOI:** 10.6026/973206300200136

**Published:** 2024-02-29

**Authors:** Pratik Surana, Kanika Singh Dhull, Ashtha Arya, Sara Samreen, Milind Rajan, Anuj Singh Parihar

**Affiliations:** 1 Department of Pedodontics and Preventive Dentistry, Maitri College of Dentistry and Research Centre, Durg, Chhattisgarh, India; 2 Department of Pedodontics & Preventive Dentistry, Kalinga Institute of Dental Sciences, KIIT Deemed to be University, Bhubaneswar, India; 3 Departmentof Conservative Dentistry and Endodontics, SGT Dental College, Hospital and Research Institute, SGT University, Gurugram, Haryana - 122505, India; 4 Department of Pedodontics and Preventive Dentistry, Maitri College of Dentistry and Research Centre, Durg, Chhattisgarh, India; 5 Department of Pedodontics and Preventive Dentistry, MA Rangoonwala Dental College, Pune - 411001, Maharashtra, India; 6 Department of Periodontology, People's Dental Academy, Bhopal, Madhya Pradesh, 462037, India

**Keywords:** Bioceramics, dentistry, applications, future considerations

## Abstract

Bioceramics have gained significant attention in dentistry due to their unique properties, including biocompatibility, osseointegration, and tissue regeneration. Therefore, it is of interest to report the various applications of bioceramics in dentistry, their advantages, limitations, and future considerations. Bio-ceramics such as zirconia and hydroxyapatite offer high biocompatibility and durability for dental application. They support bone integration for implants, resist wear, and mimic natural tooth aesthetics. Used in crowns, bridges, and fillings, bio-ceramics enhance healing and are pivotal in restorative and reconstructive dental procedures.

## Background:

The field of dentistry has witnessed remarkable advancements in materials science, leading to the development of bioceramics as a promising class of biomaterials [1]. Bioceramics primarily composed of calcium phosphates, calcium sulfates, and bioactive glasses, have emerged as valuable components for dental applications owing to their unique physical, chemical, and biological properties [2]. These materials have revolutionized various aspects of dentistry, including restorative dentistry, endodontics, periodontics, and implantology [3].

## Bioceramics:

Bioceramics are synthetic, inorganic materials that are designed to interact with biological systems. These materials possess excellent biocompatibility, bioactivity, osteo-conductivity, and potential for promoting tissue repair and regeneration [4]. Examples of commonly used bioceramics in dentistry include hydroxyapatite, tricalcium phosphate, and bioactive glasses [5]. These bioceramic materials are extensively utilized in various forms such as powders, granules, cements, and scaffolds, making them versatile for a wide range of dental applications [6].

## Rationale of using bioceramics in dentistry:

The rationale behind the use of bioceramics in dentistry stems from their unique properties that mimic the natural mineral components of the human body [1]. Bioceramics exhibit remarkable bioactivity, allowing for the formation of a strong bond with the surrounding tissues, thereby promoting osseointegration and minimizing the risk of adverse reactions [7]. Furthermore, their porous structure facilitates the ingrowth of bone, making them ideal for dental implants and bone grafting procedures [2]. The ability of bioceramics to release bioactive ions further enhances their regenerative potential, making them suitable for use in vital pulp therapy, root-end filling, and periodontal treatments [8].

## Various applications in dentistry:

Here's a detailed discussion of the application of bioceramics in dentistry:

## Dental implants:

Bioceramic materials, such as zirconia and alumina, have been widely used in the fabrication of dental implants [6]. These materials offer excellent biocompatibility, corrosion resistance, and mechanical properties, making them suitable for replacing missing teeth. Bioceramic dental implants integrate well with the jawbone, providing a stable foundation for dental prosthetics [9].

## Endodontic therapy:

Bioceramic materials, particularly calcium silicate-based bioceramics, are extensively used in root canal therapy [10]. These bioceramic sealers and root repair materials exhibit superior sealing ability, biocompatibility, and antibacterial properties, which are essential for the success of endodontic procedures [4]. Bioceramics help in filling and sealing the root canal space, preventing microbial ingress and subsequent reinfection. Additionally, these materials promote the regeneration of periapical tissues, contributing to the long-term success of root canal treatments [5].

## Dental restorations:

Bioceramic materials, such as lithium disilicate and zirconia, are widely employed in the fabrication of dental restorations, including crowns, bridges, inlays and onlays [11]. These materials exhibit excellent strength, durability, and esthetic qualities, making them ideal for producing natural-looking and long-lasting dental restorations [12].

## Periodontal treatments:

In periodontal therapy, bioceramic materials are utilized for guided tissue regeneration (GTR) and bone augmentation procedures. Bioceramic scaffolds and membranes promote the regeneration of periodontal tissues, aiding in the repair of periodontal defects and the restoration of periodontal health [12]. The biocompatibility and bio-resorbable nature of bioceramics support tissue integration and remodeling, facilitating the regeneration of periodontal structures and the stabilization of teeth affected by periodontal disease [7].

## Orthodontic applications:

Bioceramic brackets and orthodontic appliances have gained popularity in orthodontic treatments [11]. These materials offer biocompatibility, low friction, and excellent mechanical properties, providing a comfortable and efficient orthodontic experience for patients [13]. Bioceramic brackets reduce friction during tooth movement, leading to faster and more predictable orthodontic outcomes [14].

## Maxillofacial reconstruction:

Bioceramic materials play a crucial role in maxillofacial reconstructive procedures, including the fabrication of craniofacial implants, orbital implants, and bone substitutes [15]. The biocompatibility, osteoconductivity, and customizable nature of bioceramics make them suitable for restoring the form and function of craniofacial structures following traumatic injuries, congenital anomalies, or surgical interventions [16]. In summary, the application of bioceramics in dentistry encompasses a wide range of procedures and treatments, including dental implants, endodontic therapy, restorative dentistry, periodontal treatments, orthodontics, and maxillofacial reconstruction [1].

## Advantages:

Bioceramics offers several advantages in dentistry that contributes to enhanced dental procedures and outcomes [2].

## Biocompatibility:

Bioceramics exhibit excellent biocompatibility, reducing the risk of inflammatory responses and promoting tissue healing [15].

## Bioactivity:

The bioactive nature of bioceramics facilitates the formation of a strong bond with living tissues, promoting osseointegration and tissue regeneration [16].

## Osteo-conductivity:

Bioceramics provide a favorable environment for bone ingrowth and regeneration, making them suitable for bone grafting and periodontal applications [17].

## Esthetics:

Bioceramic restorations offer natural-looking and esthetic results, enhancing patient satisfaction and quality of life [10].

## Radiopacity:

Certain bioceramics have radiopacity properties, aiding in their identification on dental radiograph, which is essential for post-treatment assessment and follow-up [11].

## Limitations:

Although Bioceramics offers numerous advantages, they also have some limitations:[4].

## Mechanical strength:

Some bioceramics may exhibit lower mechanical strength compared to metal implants, limiting their use in load-bearing applications [12].

## Cost:

High-quality bioceramic materials may be more expensive than traditional dental materials, impacting their widespread adoption in dental practices. [12].

## Future considerations:

The future of bioceramics in dentistry holds immense promise, with on-going research focusing on enhancing their mechanical properties, tailoring their degradation rates, and developing advanced fabrication techniques [7]. Nanotechnology and bio-functionalization are anticipated to play a vital role in optimizing the biological response and functional performance of bioceramic materials, leading to the development of next-generation dental implants, drug delivery systems, and tissue engineering scaffolds [8].

## Conclusion:

Bioceramics have emerged as valuable biomaterials in dentistry, offering a wide array of applications and advantages in various dental specialties. Despite certain limitations, on-going research and innovations in material science continue to drive the development of advanced bioceramic materials with enhanced properties.
